# Bioinformatics and system biology approach to discover the common pathogenetic processes between COVID-19 and chronic hepatitis B

**DOI:** 10.1371/journal.pone.0323708

**Published:** 2025-05-23

**Authors:** Xiao Ma, Tengda Huang, Yujia Song, Hongyuan Pan, Ao Du, Xinyi Zhou, Yong Zeng, Kefei Yuan

**Affiliations:** Division of Liver Surgery, Department of General Surgery and Laboratory of Liver Surgery, and State Key Laboratory of Biotherapy, West China Hospital, Sichuan University, Chengdu, China; Rutgers: Rutgers The State University of New Jersey, UNITED STATES OF AMERICA

## Abstract

**Introduction:**

The ongoing coronavirus disease 2019 (COVID-19) pandemic, caused by the severe acute respiratory syndrome coronavirus 2 (SARS-CoV-2), presents a significant global public health threat. Concurrently, hepatitis B virus (HBV) remains a significant public health challenge. While previous studies have indicated an association between COVID-19 and chronic hepatitis B, the common underlying pathogenesis of these diseases remains incompletely understood.

**Methods:**

To investigate the shared molecular mechanisms between chronic HBV infection and COVID-19, a comprehensive investigation was conducted using bioinformatics and systems biology. Specifically, we utilized RNA-seq datasets (GSE196822 and GSE83148) to identify differentially expressed genes (DEGs) associated with both SARS-CoV-2 and HBV infection. Subsequently, these common DEGs were utilized to identify shared pathways, hub genes, transcriptional regulatory networks, and potential drugs. The differential expression of hub genes in both COVID-19 and HBV was verified using the GSE171110 and GSE94660 datasets, respectively.

**Results:**

From the 106 shared DEGs identified, immune-related pathways were found to play a role in the development and progression of chronic hepatitis B and COVID-19. Protein-protein interaction (PPI) network analysis revealed 8 hub genes: *CDK1*, *E2F7*, *E2F8*, *TYMS*, *KIF20A*, *CENPE*, *TPX2*, *HMMR*, *CD8A*, *GZMA*. In the validation set, the expression of hub genes was statistically significant in both the COVID-19 group and the HBV group compared with the healthy control group. Transcriptional regulatory network analysis identified 155 microRNAs (miRNAs) and 43 transcription factors (TFs) as potential regulatory signals. Notably, we identified potential therapeutic drugs for HBV chronic infection and COVID-19, including progesterone, estradiol, dasatinib, aspirin, etoposide, irinotecan hydrochloride, phorbol 12-myristate 13-acetate, lucanthone, calcitriol.

**Conclusion:**

This research elucidates potential molecular targets, signaling pathways, and promising small molecule compounds that could aid in the treatment of chronic HBV infection and COVID-19.

## Introduction

Coronavirus disease 2019 (COVID-19), resulting from the severe acute respiratory syndrome coronavirus 2 (SARS-CoV-2), has posed a tremendous challenge to the global security[1]. As of August 2023, the SARS-Cov-2 fatality rate is around 0.90%, with around 769,369,823 confirmed cases and 6,954,336 deaths. According to a World Health Organization (WHO) case report, the most prevalent symptoms of COVID-19 include fever, dry cough, sore throat, fatigue and diarrhea[2]. In addition, vomiting, headache, nausea and chest pain have been documented[3]. Angiotensin-converting enzyme 2 (ACE2) serves as a pivotal receptor for SARS-CoV-2 invasion of host cells[4]. ACE2 binds directly to the spike proteins of various coronaviruses and exhibits a high affinity for SARS-CoV-2, rendering it essential for viral entry[5]. ACE2 is abundantly expressed in the liver, bladder, heart, lungs, kidneys, and ileum[6,7]. Moreover, liver progenitor cells also express SARS-CoV-2 major cofactor transmembrane serine protease 2 (TMPRSS2)[8,9]. Several studies have identified the presence of SARS-CoV-2 RNA and protein in liver tissue from COVID-19 patients[10,11]. Patients with cirrhosis demonstrated a significantly elevated risk of death compared to cirrhosis-free patients who tested positive for SARS-CoV-2 during the same period[12]. Similarly, SARS-CoV-2 can induce abnormal liver function, liver damage, liver failure, and even death in patients with chronic Hepatitis B virus (HBV) infection[13].

Hepatitis B virus is highly prevalent, estimated to infect 300 million people worldwide, with approximately 1.5 million new infections annually[14]. Hepatitis B, a significant global health concern resulting from HBV infection, can lead to chronic infection. Currently, contact with infected patients’ body fluids and blood remains the primary transmission route for HBV[15]. The immune response is deemed the principal factor in HBV-induced liver injury, although the virus and its products may also exert cytopathogenic effects on host cells, eliciting changes in cell morphology, function, and biosynthesis that could prove fatal to the host cells[16]. During acute Hepatitis B virus infection, the majority of patients may exhibit no distinct symptoms, while a small subset may experience symptoms such as abdominal pain, nausea, vomiting, fatigue, and jaundice. In cases of long-term HBV infection, some patients may develop complications such as cirrhosis and hepatocellular carcinoma (HCC), characterized by a high incidence, poor prognosis, and elevated mortality rate[17]. Liver dysfunction observed in COVID-19 patients, including direct effects of SARS-CoV-2 on hepatocytes, cytokine storm, hypoxic-ischemic liver injury, drug-induced liver injury, and reactivation of pre-existing liver disease, has been reported as potential causes of liver injury[18–20]. To date, the potential association between Hepatitis B and COVID-19 remains incompletely understood. Thus, comprehending the interaction between COVID-19 and hepatitis B is crucial.

**Fig 1:**
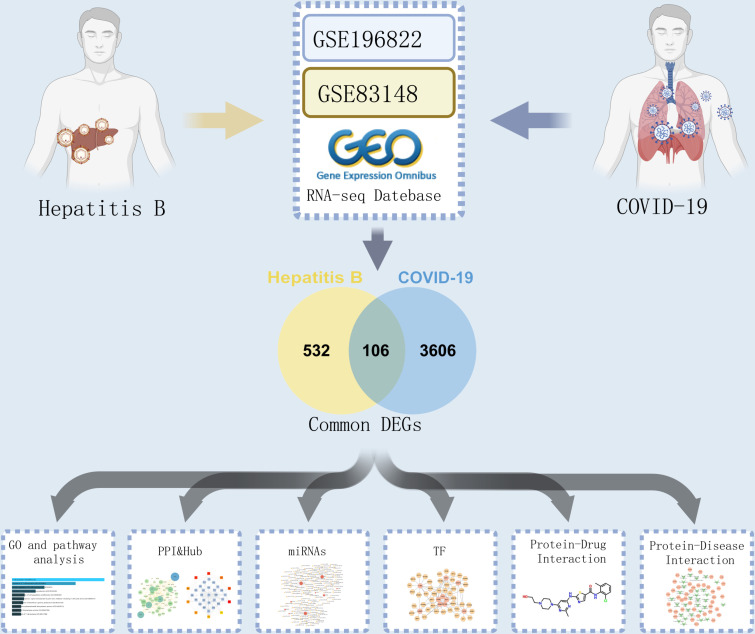
Schematic diagram of the overall general work flow of this study.

Given the COVID-19 and hepatitis B pandemics, the association between these two diseases cannot be overlooked. In this study, we obtained the COVID-19 dataset (GSE196822) and chronic hepatitis B dataset (GSE83148) from the Gene Expression Omnibus (GEO) resource. Subsequently, we identified differentially expressed genes (DEGs) and shared DEGs. Gene ontology (GO) and pathway analyses were conducted to investigate the functions of shared DEGs in these two diseases. Following protein-protein interaction analysis, we explored transcriptional regulation networks and predicted potential drugs. Moreover, we investigated the connection between hub genes and diseases. Fig 1 illustrates the main procedures of this study. This research will facilitate further exploration of common pathogenesis and suggest potential drugs for COVID-19 and hepatitis B.

## Materials and methods

### 2.1 Data collection

To elucidate the common pathogenetic processes between HBV infection and COVID-19, RNA-seq and microarray datasets from the GEO database (https://www.ncbi.nlm.nih.gov/geo/)[21] were retrieved. The COVID-19 dataset (GEO accession number: GSE196822[22,23]) comprised 43 samples, including 34 COVID-19 whole blood samples and 9 healthy controls. The GSE196822 dataset was based on high-throughput sequencing using the GPL20301 Illumina HiSeq 4000 platform (Homo sapiens) for RNA sequence extraction. The GSE171110 dataset[24] contains the whole-blood gene expression profiles of 44 COVID-19 patients and 10 healthy donors. The GSE171110 dataset was expression profiling by high throughput sequencing, which was based on GPL16791 Illumina HiSeq 2500 (Homo sapiens) for RNA sequence extraction. GSE83148[25] dataset consists of 122 HBV-infected liver tissues and 6 healthy control samples, which was based on Affymetrix Human Genome U133 Plus 2.0 Array platform. GSE94660[26] (21 paired normal and HBV-related HCC tissue samples) was expression profiling by high throughput sequencing, which was established using GPL16791 Illumina HiSeq 2500 (Homo sapiens).

### 2.2 Identification of differentially expressed genes between hepatitis B and COVID-19

The DESeq2 package[27] of R software was employed for identifying DEGs. As a comprehensive method for differential analysis of count data, DESeq2, a comprehensive method for differential analysis of count data, employs shrinkage estimators for fold change (FC) and dispersions, enhancing the stability and reproducibility of results. GEO2R (www.ncbi.nlm.nih.gov/geo/geo2r/)[21] is an online web-based tool that can be employed to compare and analyze gene expression between different sample groups. In this study, DESeq2 was used to identify DEGs for GSE196822 and we employed GEO2R to analyze DEGs for GSE83148. Genes demonstrating p-value* *< 0.05 combined with |log_2_ FC| > 1 were identified as statistically significant DEGs. To identify common DEGs from these two datasets, all identified DEGs were imported into JVenn (http://jvenn.toulouse.inra.fr/app/example.html)[28], an online tool that enables comparison of gene lists irrespective of the methods and conditions used in different studies. Upon uploading the DEG lists, JVenn generates Venn diagrams that visually represent the shared DEGs. To assess the statistical significance of the overlap of DEGs between COVID-19 and HBV, Fisher’s Exact Test was performed. This method uses a 2 × 2 contingency table to calculate the p-value and determines whether the observed overlap significantly differs from random overlap.

### 2.3 Gene ontology and pathway enrichment analysis

Enrichment analysis, a popular method for analyzing gene sets from genome-wide experiments, has proven valuable in life sciences. The gene ontology (GO) resource, aiming to provide a broad, structured computational model of biological systems, has emerged as the largest collection of information regarding gene and gene product functions. The GO analysis was then subdivided into three groups: biological process (BP), cellular component (CC), and molecular function (MF) of common DEGs. To unveil potential molecular pathways, the BioCarta, Kyoto Encyclopedia of Genes and Genomes (KEGG), Reactome and WikiPathways were employed. BioCarta offers insights into molecular pathways and cellular processes. KEGG provides detailed information on metabolic pathways, cellular mechanisms, and disease-related pathways. Reactome, an open-source database, focuses on biological reactions and molecular pathways, making it suitable for systems biology research. WikiPathways is a community-driven database that supports the study of various biological processes through its continuously updated pathways. Enrichr (https://maayanlab.cloud/Enrichr/)[29], facilitating both a comprehensive resource for curated gene sets and a browser accumulating biological information, was utilized for all mentioned GO and pathways analyses. Results were considered statistically significant when the p-value was < 0.05.

### 2.4 Protein–protein interactions (PPI) analysis and hub gene extraction

Biological processes are typically executed by groups of interacting proteins. By using proteins expressed by the common DEGs from HBV and COVID-19 datasets, a protein-protein interaction network was established to clarify crucial principles of protein organizations. In this study, PPIs were constructed using the STRING databank (https://string-db.org/)[30]. The PPI network was then conducted and investigated using Cytoscape (version 3.7.1). Cytohubba[31] (https://apps.cytoscape.org/apps/cytohubba), a Cytoscape plug-in, was eventually used to evaluate and rank the top 10 hub genes, representing the most entangled and prominent nodes in the PPI network. Maximum Connected Component (MCC)[32] was used to filter and identify subnetworks that have important functions in molecular interaction networks, leading to a better understanding of protein interactions and their impact on biological processes.

### 2.5 Regulatory analysis of the hub gene

Transcription factors (TFs) are proteins that precisely modulate gene expression by regulating the transcription process and then determine organism phenotypes accordingly. MicroRNAs (miRNAs) are short noncoding RNAs that can mediate gene silencing and regulate protein synthesis on the post-transcriptional stage. These factors contribute to a complex network essential for gene regulation. The JASPAR database(http://jaspar.genereg.net/)[33] was also applied in this study to discover gene-TF-interaction networks. Moreover, to explore gene-miRNA-interaction networks, the miRTarBase (http://miRTarBase.cuhk.edu.cn/) database was put into practice[34].

### 2.6 Exploration of candidate drugs

Drug Signatures Database (DSigDB) is a resource that combines drugs with target genes[35]. Founded on the principles of quantitative inhibition and mechanisms of drug-induced gene expression, DSigDB can give a reference for targeted medicines and specific treatments of diseases. In this study, the DSigDB was used to forecast protein–drug interrelationships and reveal candidate compounds that may interact with the hub genes. A metric of p-value <0.05 was set up to determine the top functional compounds and pathways.

### 2.7 Gene-disease association analysis

As more and more genes and variants were discovered, it is of great importance to identify the clinical significance of these loci. Therefore, DisGeNET (http://www.disgenet.org/), a knowledge platform that collects disease-associated genes and their variants, was created to combine the results with the latest findings[36]. In this study, NetworkAnalyst (https://www.networkanalyst.ca/) was used to analyze gene-disease interrelationships in order to reveal symptoms and diseases connected with the hub genes.

## Results

### 3.1 Identification of common DEGs between HBV and COVID-19

**Fig 2:**
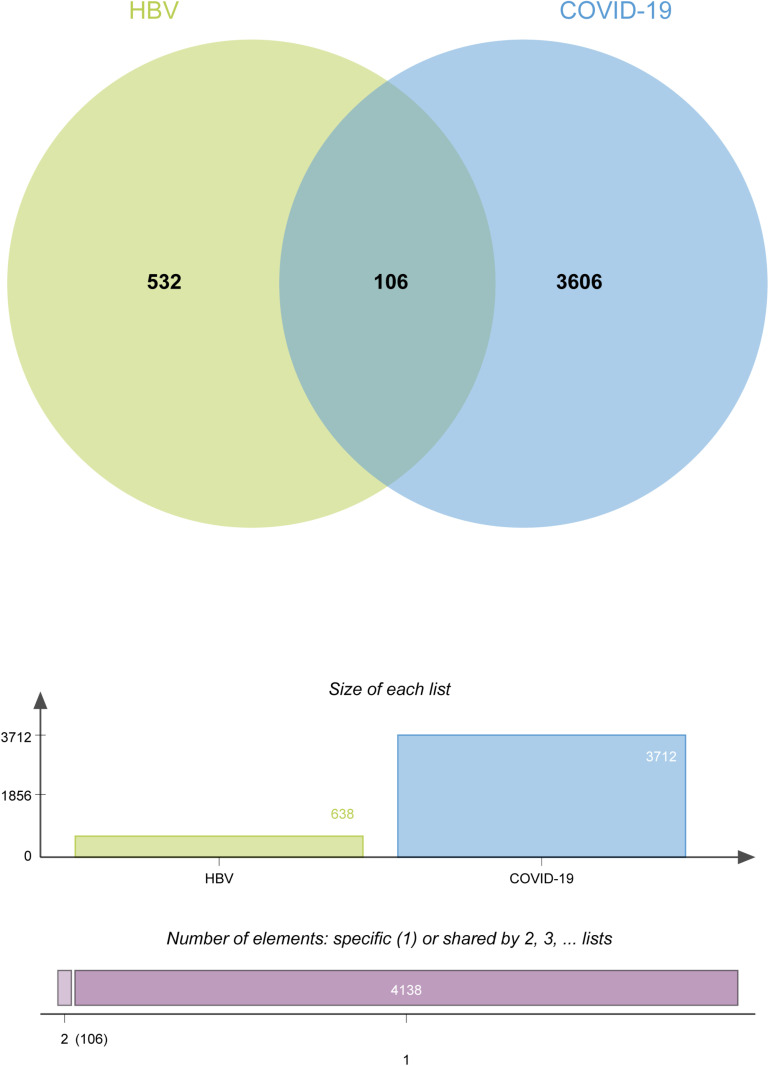
The Venn diagram showed 106 common DEGs of HBV and COVID-19. (p-value = 6.52e-09).

To reveal the underlying interrelationships between HBV and COVID-19, the microarray and high throughput sequenced datasets were acquired from GEO. In the GSE83148 dataset, 638 DEGs were identified, of which 582 were upregulated and 56 were downregulated (S1 Table). Similarly, in the GSE196822 dataset, 3,734 DEGs were recognized, of which 1,310 were upregulated and 2,424 were downregulated (S2 Table). The summarized information of DEGs is listed in Table 1. Next, all the identified DEGs above were then uploaded to Jvenn, which generates a Venn diagram. After that, we found 106 common DEGs, which were investigated subsequently (Fig 2 and S3 Table). Fisher’s Exact Test revealed a highly significant p-value (p-value = 6.52e-09), indicating a statistically significant difference between the observed DEG overlap and random overlap. By identifying common DEGs between the two diseases, the link between them can be clarified through further analysis.

**Table 1:** Overview of the datasets in this analysis.

Disease name	GEO accession	GEO platform	Total DEGs count	Upregulated DEGs count	Downregulated DEGs count
COVID-19	GES196822	GPL20301	3,734	1,310	2,424
HBV	GSE83148	GPL570	638	582	56

### 3.2 Gene ontology and pathway enrichment analysis

**Fig 3:**
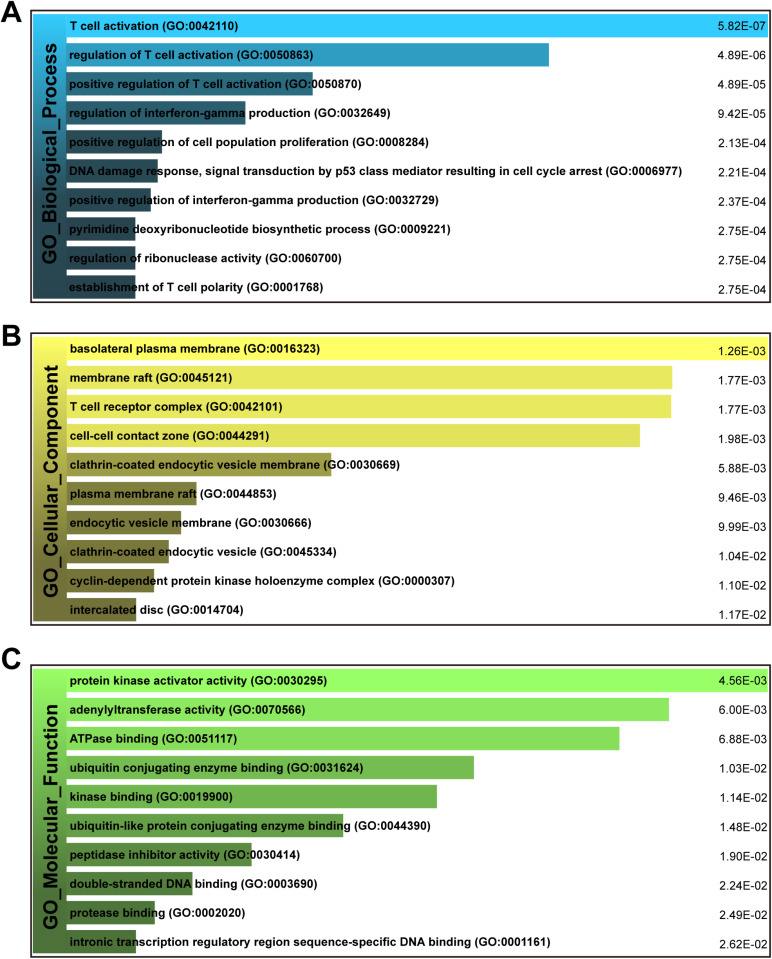
The bar chart of the GO assessment of the shared DEGs associated with HBV and COVID-19. (A) biological processes, (B) cellular component, and (C) molecular function.

The GO database is a universally recognized resource representing the functions of genes and gene products. Fig 3A–3C present the results of the GO enrichment analysis in the form of bar graphs. Table 2 listed the top 10 terms of biological process, molecular function and cellular component category. Notably, the 106 common DEGs significantly enriched in immune-related pathways, including T cell activation (GO:0042110), regulation of T cell activation (GO:0050863), positive regulation of T cell activation (GO:0050870), regulation of interferon-gamma production (GO:0032649), positive regulation of interferon-gamma production (GO:0032729), establishment of T cell polarity (GO:0001768) and T cell receptor complex (GO:0042101).

**Table 2:** Ontological analysis of common DEGs between HBV and COVID-19.

Category	GO ID	Term	p-value	Genes
GO biological process	GO:0042110	T cell activation	5.82E-07	CD2; ITK; CD8A; CRTAM; LEPR; CCR7; RASGRP1
	GO:0050863	regulation of T cell activation	4.89E-06	CD2; SIRPG; CRTAM; HLA-DPB1; PRKCQ
	GO:0050870	positive regulation of T cell activation	4.89E-05	SIRPG; HLA-DPB1; FLOT2; CCR7; PRKCQ
	GO:0032649	regulation of interferon-gamma production	9.42E-05	CD2; CRTAM; HLA-DPB1; CCR7; RASGRP1
	GO:0008284	positive regulation of cell population proliferation	2.13E-04	CDKN1A; EPCAM; PDGFD; SIRPG; RHOG; TCIRG1; TIMP1; ZFPM2; TOX; EGFR
	GO:0006977	DNA damage response, signal transduction by p53 class mediator resulting in cell cycle arrest	2.21E-04	CDKN1A; CDK1; E2F7; E2F8
	GO:0032729	positive regulation of interferon-gamma production	2.37E-04	CD2; CRTAM; HLA-DPB1; RASGRP1
	GO:0009221	pyrimidine deoxyribonucleotide biosynthetic process	2.75E-04	CMPK2; TYMS
	GO:0060700	regulation of ribonuclease activity	2.75E-04	OAS2; OAS3
	GO:0001768	establishment of T cell polarity	2.75E-04	CRTAM; CCR7
GO cellular component	GO:0016323	basolateral plasma membrane	1.26E-03	SLC38A1; EPCAM; FLOT2; ANK2; EGFR
	GO:0045121	membrane raft	1.77E-03	CD8A; CD24; CTSD; MS4A1; EGFR
	GO:0042101	T cell receptor complex	1.77E-03	CD8A; CD3D
	GO:0044291	cell-cell contact zone	1.98E-03	JUP; FLOT2; ANK2
	GO:0030669	clathrin-coated endocytic vesicle membrane	5.88E-03	HLA-DPB1; CD3D; EGFR
	GO:0044853	plasma membrane raft	9.46E-03	CD8A; FLOT2; MS4A1
	GO:0030666	endocytic vesicle membrane	9.99E-03	HLA-DPB1; TCIRG1; CD3D; EGFR
	GO:0045334	clathrin-coated endocytic vesicle	1.04E-02	HLA-DPB1; CD3D; EGFR
	GO:0000307	cyclin-dependent protein kinase holoenzyme complex	1.10E-02	CDKN1A; CDK1
	GO:0014704	intercalated disc	1.17E-02	JUP; ANK2
GO molecular function	GO:0030295	protein kinase activator activity	4.56E-03	TPX2; CDKN1A; CD24
	GO:0070566	adenylyltransferase activity	6.00E-03	OAS2; OAS3
	GO:0051117	ATPase binding	6.88E-03	ANK2; TCIRG1; EGFR
	GO:0031624	ubiquitin conjugating enzyme binding	1.03E-02	RNF125; SIAH2
	GO:0019900	kinase binding	1.14E-02	TPX2; CDKN1A; RHOG; ANK2; KIF20A; CD24; EGFR
	GO:0044390	ubiquitin-like protein conjugating enzyme binding	1.48E-02	RNF125; SIAH2
	GO:0030414	peptidase inhibitor activity	1.90E-02	TIMP1; CST7
	GO:0003690	double-stranded DNA binding	2.24E-02	EOMES; BCL11B; PYHIN1; NR1D2; TEAD2; EGFR; E2F7; E2F8
	GO:0002020	protease binding	2.49E-02	ECM1; COL1A2; TIMP1
	GO:0001161	intronic transcription regulatory region sequence-specific DNA binding	2.62E-02	GRHL2

**Fig 4:**
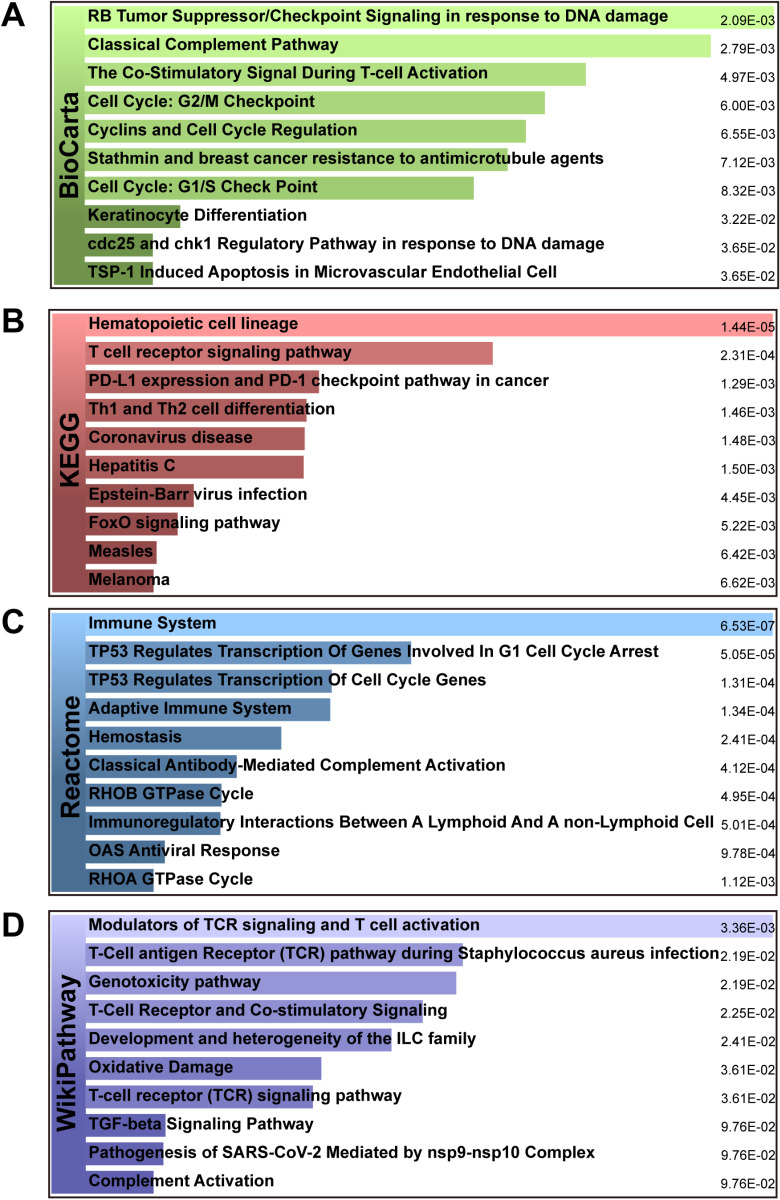
The bar graphs of the pathway enrichment of the shared DEGs between HBV and COVID-19. (A) BioCarta, (B) KEGG, (C) Reactome, and (D) WikiPathways.

In the pathway analysis, we used four databases, including BioCarta, KEGG, Reactome, and WikiPathway, to analyze the function of shared DEGs. Table 3 depicts the top enriched pathways in different databases. The details of the pathway enrichment analysis were shown in Fig 4A–4D. From the results of the BioCarta enrichment analysis, these 106 shared DEGs were mainly enriched in the regulation of cell cycle and T-cell activation, including the co-stimulatory signal during T-cell activation, cell cycle: G2/M checkpoint, cyclins and cell cycle Regulation and cell cycle: G1/S check point. Most importantly, the KEGG analysis indicated that common DEGs may affect the progression of various infectious diseases and cancer, including coronavirus disease, hepatitis C, Epstein-Barr virus infection, FoxO signaling pathway, measles and melanoma. Besides, the Reactome analysis also showed that mutual DEGs were significantly enriched in antiviral, cell cycle and immune-related pathways, including immune system, TP53 regulates transcription of cell cycle genes, immunoregulatory interactions between A lymphoid and A non-Lymphoid cell and OAS antiviral response. Similarly, WikiPathways analysis also found that mutual DEGs affected tumour progression and immune-related pathways, including modulators of TCR signaling and T cell activation, T-Cell receptor and co-stimulatory signaling, T-cell receptor (TCR) signaling pathway and TGF-beta signaling pathway. Thus, these common DEGs affect the disease progression of HBV and COVID-19 through immune-related pathways.

**Table 3:** Pathway enrichment analysis of common DEGs between HBV and COVID-19.

Category	Pathways	p-value	Genes
BioCarta	RB Tumor Suppressor/Checkpoint Signaling in response to DNA damage	2.09E-03	CDKN1A; CDK1
	Classical Complement Pathway	2.79E-03	C1QB; C1QC
	The Co-Stimulatory Signal During T-cell Activation	4.97E-03	ITK; CD3D
	Cell Cycle: G2/M Checkpoint	6.00E-03	CDKN1A; CDK1
	Cyclins and Cell Cycle Regulation	6.55E-03	CDKN1A; CDK1
	Stathmin and breast cancer resistance to antimicrotubule agents	7.12E-03	CD2; CDK1
	Cell Cycle: G1/S Check Point	8.32E-03	CDKN1A; CDK1
	Keratinocyte Differentiation	3.22E-02	PRKCQ; EGFR
	cdc25 and chk1 Regulatory Pathway in response to DNA damage	3.65E-02	CDK1
	TSP-1 Induced Apoptosis in Microvascular Endothelial Cell	3.65E-02	GZMA
KEGG	Hematopoietic cell lineage	1.44E-05	CD2; CD8A; HLA-DPB1; CD24; CD3D; MS4A1
	T cell receptor signaling pathway	2.31E-04	ITK; CD8A; PRKCQ; RASGRP1; CD3D
	PD-L1 expression and PD-1 checkpoint pathway in cancer	1.29E-03	PRKCQ; CD3D; RASGRP1; EGFR
	Th1 and Th2 cell differentiation	1.46E-03	STAT4; HLA-DPB1; PRKCQ; CD3D
	Coronavirus disease	1.48E-03	C1QB; OAS2; OAS3; MX2; EGFR; C1QC
	Hepatitis C	1.50E-03	CDKN1A; OAS2; OAS3; MX2; EGFR
	Epstein-Barr virus infection	4.45E-03	CDKN1A; OAS2; OAS3; HLA-DPB1; CD3D
	FoxO signaling pathway	5.22E-03	CDKN1A; FBXO32; SGK1; EGFR
	Measles	6.42E-03	OAS2; OAS3; MX2; CD3D
	Melanoma	6.62E-03	CDKN1A; PDGFD; EGFR
Reactome	Immune System R-HSA-168256	6.53E-07	C1QB; ITK; CDKN1A; KLRB1; TCIRG1; RASGRP1; CD3D; STAT4; TIMP1; CTSD; JUP; BCL11B; SIAH2; MX2; CRTAM; SH2D1A; RHOG; FBXO32; CKAP4; CENPE; RNF125; COL1A2; CD8A; OAS2; OAS3; PRKCQ; KIF20A; C1QC
	TP53 Regulates Transcription Of Genes Involved In G1 Cell Cycle Arrest R-HSA-6804116	5.05E-05	CDKN1A; E2F7; E2F8
	TP53 Regulates Transcription Of Cell Cycle Genes R-HSA-6791312	1.31E-04	CDKN1A; CDK1; E2F7; E2F8
	Adaptive Immune System R-HSA-1280218	1.34E-04	ITK; KLRB1; SIAH2; CRTAM; SH2D1A; FBXO32; RASGRP1; CD3D; CENPE; CD8A; PRKCQ; KIF20A; CTSD
	Hemostasis R-HSA-109582	2.41E-04	CD2; CENPE; ECM1; EPCAM; SIRPG; RHOG; PRKCQ; KIF20A; TIMP1; ZFPM2; RASGRP1
	Classical Antibody-Mediated Complement Activation R-HSA-173623	4.12E-04	C1QB; C1QC
	RHOB GTPase Cycle R-HSA-9013026	4.95E-04	JUP; MCAM; ARHGEF3; FLOT2
	Immunoregulatory Interactions Between A Lymphoid And A non-Lymphoid Cell R-HSA-198933	5.01E-04	KLRB1; CD8A; CRTAM; SH2D1A; CD3D
	OAS Antiviral Response R-HSA-8983711	9.78E-04	OAS2; OAS3
	RHOA GTPase Cycle R-HSA-8980692	1.12E-03	PGRMC2; JUP; MCAM; ARHGEF3; FLOT2
WikiPathway	Modulators of TCR signaling and T cell activation WP5072	3.36E-03	ITK; CD8A; SH2D1A; PRKCQ; CD3D
	T-Cell antigen Receptor (TCR) pathway during Staphylococcus aureus infection WP3863	2.19E-02	ITK; CD8A; PRKCQ; CD3D
	Genotoxicity pathway WP4286	2.19E-02	CENPE; CDKN1A; E2F7; E2F8
	T-Cell Receptor and Co-stimulatory Signaling WP2583	2.25E-02	ITK; CD8A; RASGRP1
	Development and heterogeneity of the ILC family WP3893	2.41E-02	EOMES; BCL11B; TOX
	Oxidative Damage WP3941	3.61E-02	C1QB; CDKN1A; C1QC
	T-cell receptor (TCR) signaling pathway WP69	3.61E-02	ITK; CD8A; PRKCQ; CD3D
	TGF-beta Signaling Pathway WP366	9.76E-02	CDKN1A; COL1A2; STAMBPL1; CDK1
	Pathogenesis of SARS-CoV-2 Mediated by nsp9-nsp10 Complex WP4884	9.76E-02	CD2; CD8A
	Complement Activation WP545	9.76E-02	C1QB; C1QC

### 3.3 PPI analysis and extraction of hub genes

**Fig 5:**
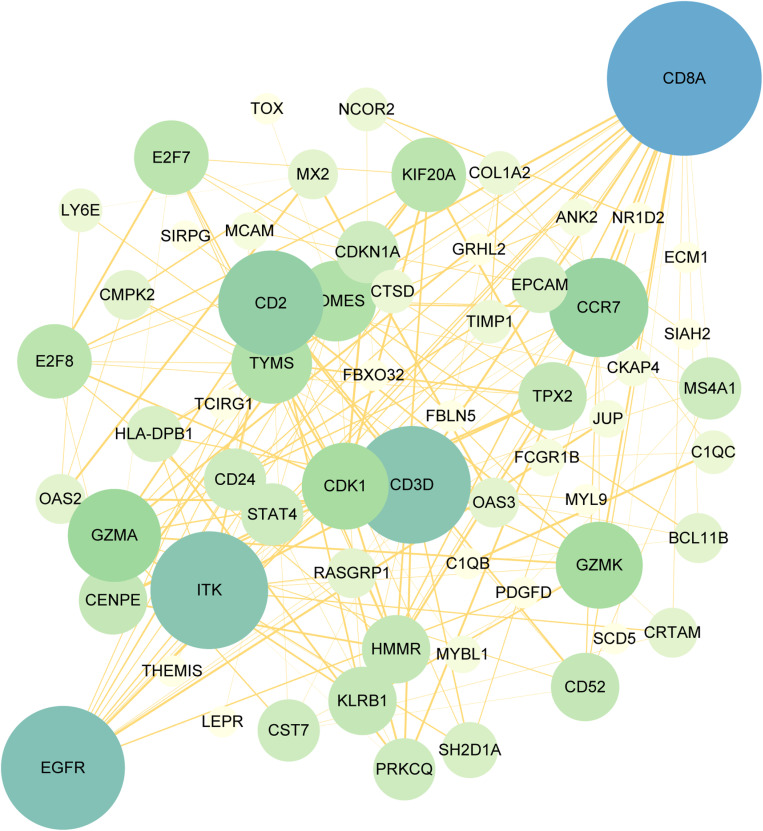
PPI network of the mutual DEGs between COVID-19 and HBV. The nodes and the edges of the figure represent DEGs and the interactions between the nodes, respectively. The PPI network contains 104 nodes and 166 edges. The size and color depth of the circle indicate the extent to which proteins are connected.

**Fig 6:**
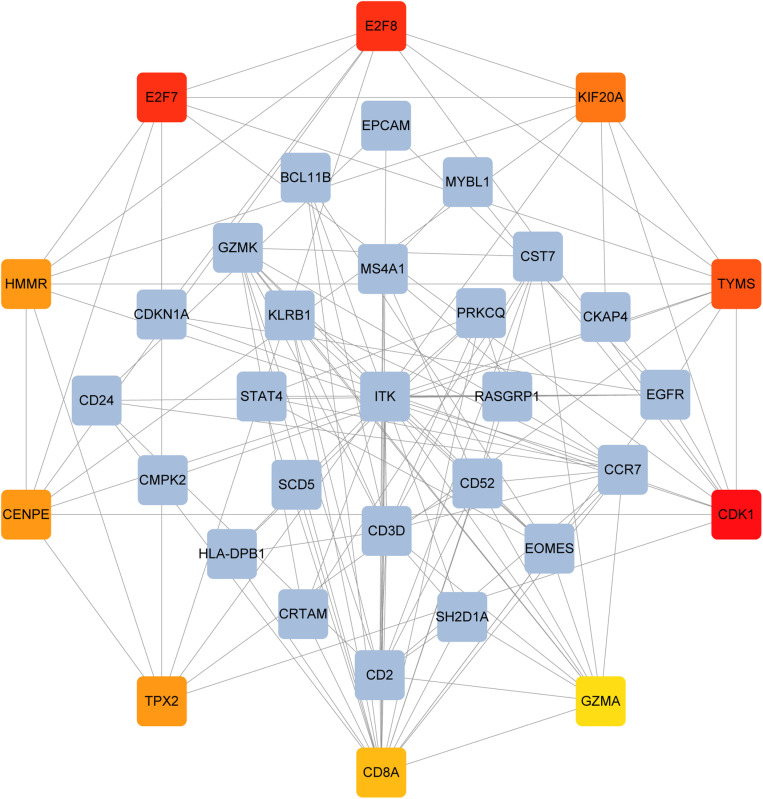
PPI network from all the shared DEGs is constructed by Cytohubba plugin in Cytosacpe. Red nodes present the selected top 10 hub genes. The network has 35 nodes and 126 edges. Their interactions with other molecules are shown by the red, orange, and yellow nodes (their rank was indicated by a gradient from red to yellow).

**Fig 7:**
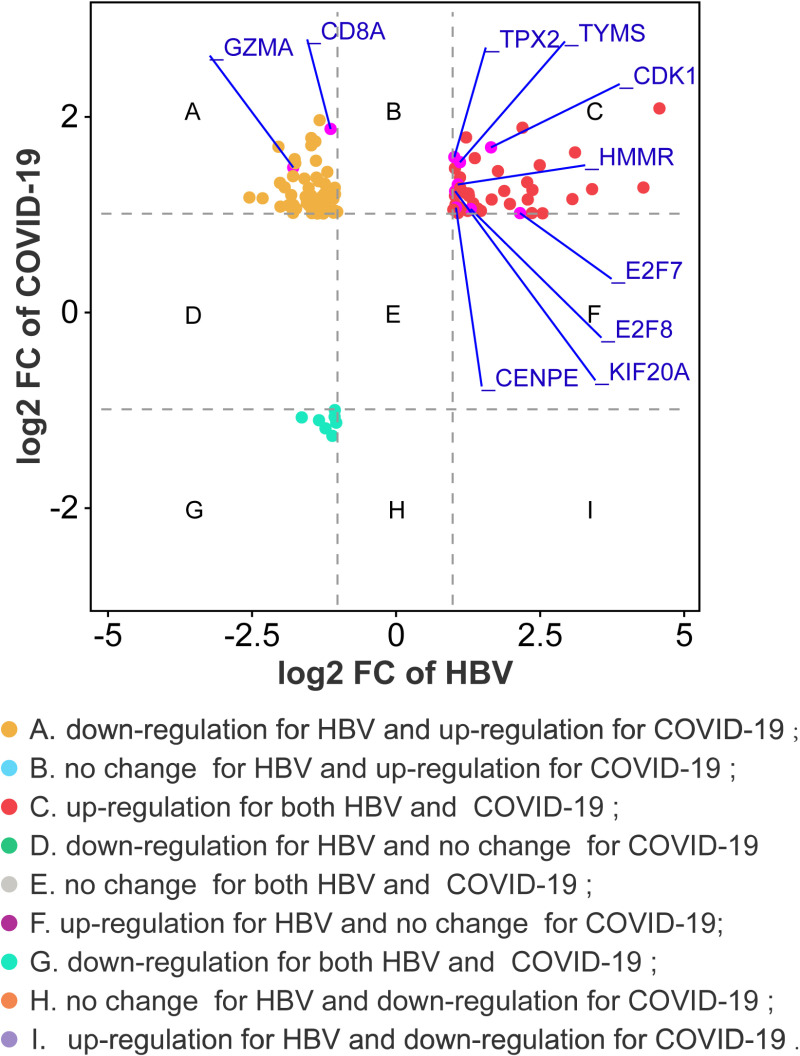
Fold changes of COVID-19 and PC at transcriptional level. Nine categories in different colors indicate nine responsive groups (|log_2_ Fold Change| ≥ 1 and *P* value<0.05).

**Fig 8:**
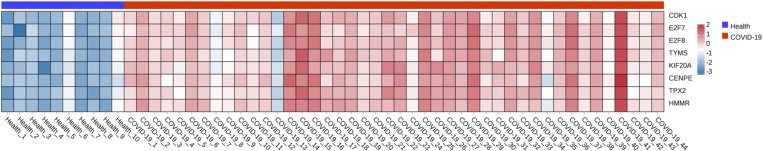
Heatmap of hub genes expression abundance in the validation set GSE171110.

**Fig 9:**
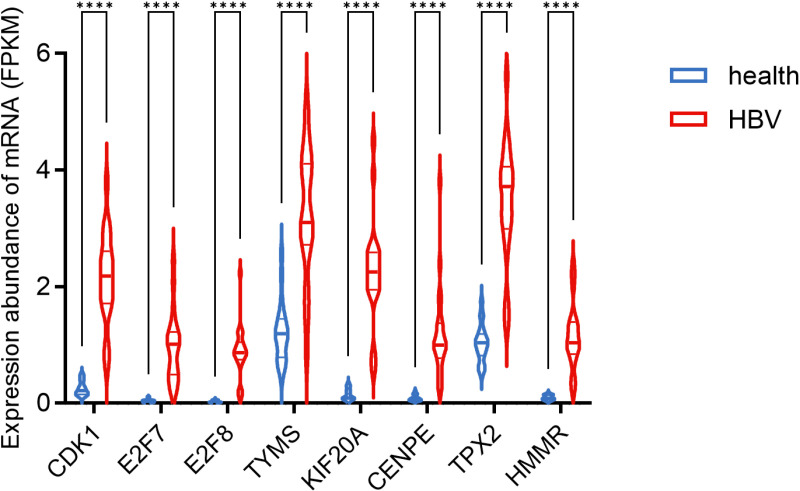
The expression of hub genes in the GSE94660 dataset.

A protein-protein network was employed to uncover the interactions of shared DEGs, composed of nodes and edges. The PPI network of common DEGs included 104 nodes and 166 edges and was demonstrated in Fig 5. Next, cytoHubba, one of Cytoscapes’s numerous plugins, was used to determine the top 10 candidate hub genes, which were the most entangled nodes in the network (Fig 6). The top 10 candidate hub genes ranked by MCC method were CDK1, E2F7, E2F8, TYMS, KIF20A, CENPE, TPX2, HMMR, CD8A, GZMA. As shown in Fig 7, we further analyzed the differential expression of the shared DEGs in HBV and COVID-19, especially focusing on the differential expression of the 10 candidate hub genes. Among the ten candidate hub genes, we found that 8 hub genes were differentially upregulated in HBV and COVID-19, so we further narrowed the scope of the study to 8 hub genes, including *CDK1, E2F7, E2F8, TYMS, KIF20A, CENPE, TPX2, HMMR*. In addition, in order to ensure the repeatability of this study, we introduced two external data sets (GSE171110 and GSE94660) to verify the expression levels of 8 hub genes, as shown in Fig 8 and Fig 9. Thus, these 8 hub genes could serve as potential biomarkers and might contribute to new therapeutic strategies.

### 3.4 Gene-regulatory network analysis

**Fig 10:**
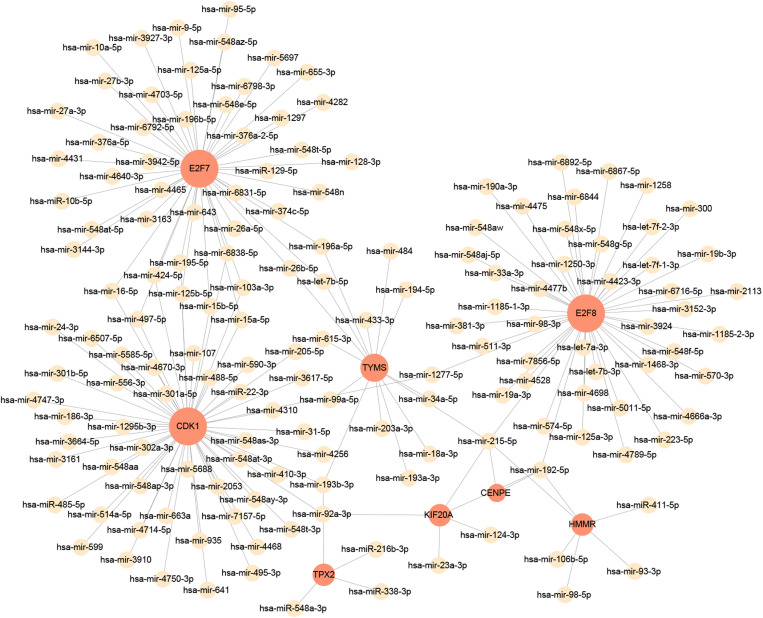
The regulatory interaction network of genes-miRNAs. MiRNAs is presented by the square node and gene symbols interacting with miRNAs are in circle. The network contains 163 nodes and 178 edges.

**Fig 11:**
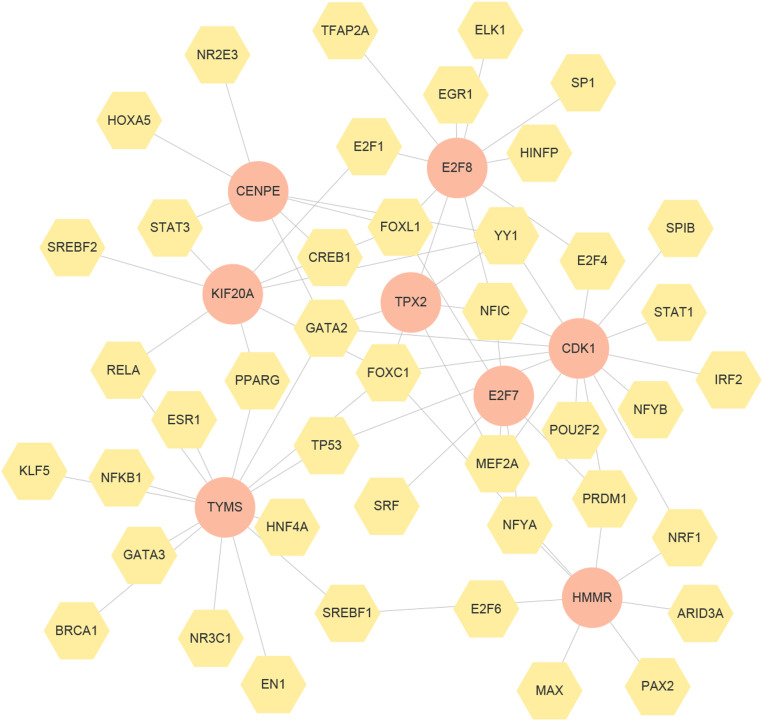
The genes-TFs interaction network created by the NetworkAnalyst. The circle nodes represent gene symbols interacting with TFs while the hexagon nodes represent TFs. The network contains 51 nodes and 71 edges.

miRNAs can regulate gene expression in the post-transcriptional stage, while TF can control genes at the transcriptional level[37]. Both miRNAs and TFs form a complicated regulatory network and play an important role in the process of gene expression[38]. Fig 10 depicts the hub gene-miRNA interaction network. Fig 11 clarify the hub gene-TF interaction network. In the miRNAs and TFs gene-regulatory network analysis, 155 miRNAs and 43 TFs regulatory signatures were expected to modulate the identified hub genes, reflecting close contacts between them. Among the identified miRNAs, key regulators such as hsa-miR-192-5p, hsa-miR-215-5p, hsa-let-7b-5p, hsa-mir-26b-5p, hsa-mir-26a-5p, and hsa-mir-98-5p were found to play crucial roles in modulating the hub genes. Similarly, transcription factors including NF-κB, STAT3, and HNF4A were identified as significant regulators within the network, suggesting their involvement in the regulatory mechanisms of the hub genes.

### 3.5 Exploration of candidate drugs

The understanding of target-drug interplay can aid in finding and developing new drugs. DSigDB is a resource that relates compounds with target genes. It was used to forecast the compounds interacting with common DEGs. During the process of analysis, we ranked the top 10 candidate drugs according to their p-values. Table 4 was created to present the selected drugs which may be possible for treating HBV and COVID-19 (progesterone, estradiol, dasatinib, aspirin, etoposide, irinotecan hydrochloride, phorbol 12-myristate 13-acetate, lucanthone, calcitriol).

**Table 4:** Potential drugs for COVID-19 and HBV.

Name	p*-*value	Chemical Formula
Progesterone CTD 00006624	2.41E-11	C_21_H_30_O_2_
Estradiol CTD 00005920	5.03E-08	C_18_H_24_O_2_
Dasatinib CTD 00004330	6.57E-08	C_22_H_26_ClN_7_O_2_S
Aspirin CTD 00005447	1.60E-06	C_9_H_8_O_4_
Etoposide MCF7 DOWN	5.43E-06	C_29_H_32_O_13_
Irinotecan hydrochloride CTD 00002224	9.34E-06	C_33_H_39_ClN_4_O_6_
Phorbol 12-myristate 13-acetate CTD 00006852	9.88E-06	C_36_H_56_O_8_
Lucanthone CTD 00006227	1.85E-05	C_20_H_24_N_2_OS
Calcitriol CTD 00005558	2.53E-05	C_27_H_44_O_3_

### 3.6 Gene-disease association analysis

**Fig 12:**
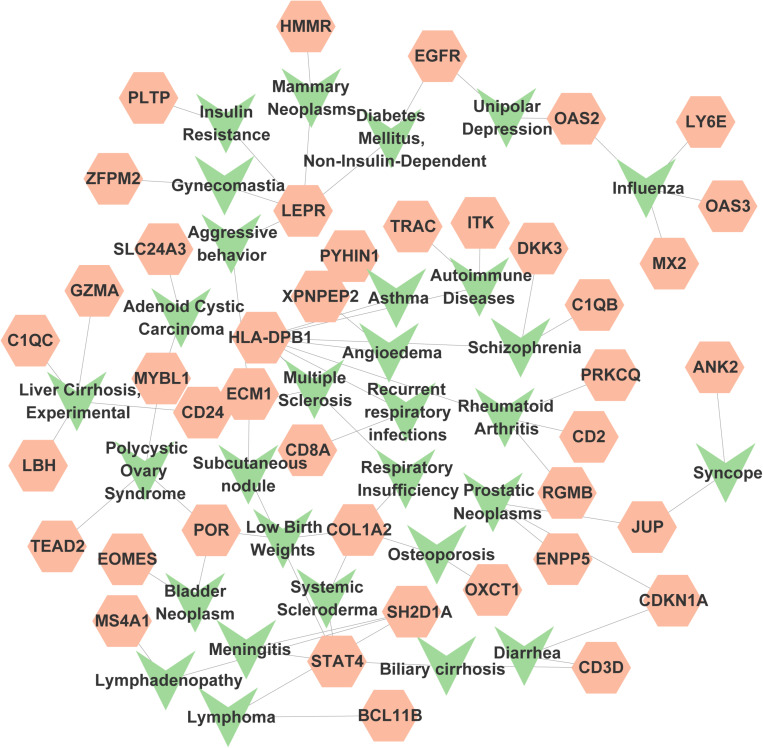
The gene-disease relationship network represents diseases associated with common DEGs. Herein, green represents diseases and orange represents genes. The network contains 71 nodes and 70 edges.

A similar set of genes can involve in molecular pathways, governing human diseases by forming sophisticated circuits and intricate networks. Study of the relationships between common genes and diseases may be very helpful in predicting the prognosis of human diseases and designing treatments. To collect the information on the relevant genes, DisGeNET was taken into consideration. DisGeNET is one of the biggest resources of genes and variants that participated in human diseases. NetworkAnalyst was then used to investigate the gene-disease association. The results indicated that recurrent respiratory infections, mammary neoplasms, lymphadenopathy, subcutaneous nodules, unipolar depression, syncope, systemic scleroderma, schizophrenia, respiratory insufficiency, prostatic neoplasms and COVID-19 were most related to the identified common DEGs. Fig 12 shows the association between genes and diseases.

## Discussion

Due to the high incidence of COVID-19 and HBV, both caused by viral pathogens, concerns arise regarding the potential exacerbation of the clinical course of COVID-19 by HBV and vice versa. Consequently, we aim to explore the co-pathogenic processes between COVID-19 and HBV utilizing systems biology and bioinformatics methods.

In this study, we identified 106 shared DEGs for COVID-19 and HBV. The pathway-level insights into the shared DEGs offer a deeper understanding of the molecular mechanisms driving both diseases. The GO analysis revealed significant enrichment in immune-related pathways, including T cell activation, regulation of interferon-gamma production, and T cell receptor complex formation. These findings suggest that immune dysregulation plays a central role in both COVID-19 and HBV pathogenesis[39]. Furthermore, pathway analysis using BioCarta, KEGG, Reactome, and WikiPathways highlighted the involvement of cell cycle regulation, antiviral responses, and immune system processes. The DEGs were notably enriched in pathways such as T cell activation, co-stimulatory signaling during T-cell activation, and cell cycle regulation (e.g., G1/S and G2/M checkpoints) according to BioCarta. This underscores the critical role of cell cycle and immune regulation in the disease progression of both infections[40]. KEGG analysis also linked these DEGs to viral infections such as coronavirus disease and hepatitis C, further emphasizing the relevance of these pathways in both diseases[41]. Reactome and WikiPathways analyses similarly highlighted immune and cell cycle-related pathways, including TP53 regulation of cell cycle genes and antiviral response pathways[42]. These pathway-level insights suggest that therapeutic strategies targeting immune regulation and cell cycle pathways could be beneficial in managing both COVID-19 and HBV.

Subsequently, a PPI network of common DEGs was constructed, revealing 8 hub genes: *CDK1, E2F7, E2F8, TYMS, KIF20A, CENPE, TPX2, HMMR*. These genes are closely associated with the pathological mechanisms underlying both diseases. CDK1 (Cyclin Dependent Kinase 1) plays a critical role in cell cycle control. It phosphorylates human telomerase reverse transcriptase (hTERT), which is essential for the transcription and replication of viral RNA after SARS-CoV-2 enters the host cell[43]. In addition, CDK1 regulates HBV replication[44] and can interact with HBV-encoded circRNA to regulate HBV-mediated hepatocellular carcinoma progression[45]. E2F7 and E2F8, members of the atypical E2F transcription factor family, primarily function as transcriptional repressors. They are key regulators of the cell cycle, inhibiting E2F target genes involved in DNA replication and repair. Dysregulation of these genes is linked to HBV-induced liver carcinogenesis[46]. SARS-CoV-2 also exploits the host cell cycle machinery to facilitate viral replication, suggesting that E2F7 and E2F8 may play a role in COVID-19 pathogenesis by modulating cell cycle and DNA repair pathways[47,48]. TYMS (Thymidylate Synthetase) is essential for DNA replication and repair. Several studies have reported that TYMS is associated with HBV replication and serves as a key gene for the prognosis and diagnosis of HBV-associated hepatocellular carcinoma[49–51]. Similarly, TYMS has been identified as a potential therapeutic target in several COVID-19 related reports[52–54]. KIF20A (Kinesin Family Member 20A), involved in intracellular transport and cell division, has been implicated in both SARS-CoV-2 [55] and HBV infection[56,57]. CENPE (Centromere Protein E) is a motor protein essential for chromosome alignment during mitosis, which ensures genomic stability. Although its direct role in HBV infection remains unexplored, genomic instability driven by HBV-related carcinogenesis may involve CENPE[58]. In the context of COVID-19, studies have reported the impact of SARS-CoV-2 on mitotic processes, suggesting a potential link between CENPE activity and viral replication or host immune response[59]. TPX2 (TPX2 Microtubule Nucleation Factor) is a microtubule-associated protein critical for mitotic spindle assembly. TPX2 plays a crucial role in HBV-related HCC and is associated with poor prognosis in patients. It contributes to HBV-induced HCC progression by regulating cell cycle and microtubule formation. Moreover, TPX2-related ceRNA networks, such as the TRHDE-AS1/miR-23b/PKIA axis, may influence HBV-related HCC pathogenesis and prognosis[60]. In COVID-19, TPX2 has been identified as a potential therapeutic target, given its role in regulating cell cycle and stress responses under viral infection[61]. HMMR (Hyaluronan-mediated Motility Receptor) plays a pivotal role in HBV-related HCC by modulating the interplay between endoplasmic reticulum (ER) stress and autophagy. Under ER stress induced by HBV, HMMR expression is transcriptionally regulated by CHOP and dynamically degraded by TRIM29. HMMR alleviates ER stress by enhancing autophagic lysosome activity, which contributes to HBV-related carcinogenesis. These findings position HMMR as a potential therapeutic target[62].

In addition to identifying the hub genes between COVID-19 and chronic hepatitis B, we further investigated the upstream regulators that could potentially influence these hub genes, specifically focusing on miRNAs and transcription factors. A total of 155 miRNAs and 43 transcription factors were identified as potential upstream regulators of the hub genes shared between these two diseases. Understanding the functional involvement of these regulatory elements is crucial for elucidating the molecular pathways underlying the pathogenesis of COVID-19 and chronic hepatitis B. MicroRNAs play a key role in the post-transcriptional regulation of gene expression by binding to the 3’ untranslated regions (UTRs) of target mRNAs, leading to mRNA degradation or translational repression. Many of the identified miRNAs have been implicated in immune response modulation, inflammatory signaling, and cell cycle regulation, all of which are central to the progression of both COVID-19 and HBV infection. For example, miRNAs such as hsa-mir-192-5p[63] and hsa-mir-215-5p[64], which are closely associated with liver function and fibrosis processes, may play important roles in HBV infection and COVID-19-related liver damage. Additionally, hsa-let-7b-5p, known for its role in regulating inflammatory responses and viral replication, is a significant target in studies on SARS-CoV-2 and other viruses[65,66]. hsa-mir-26b-5p[67] and hsa-mir-26a-5p[68] are thought to modulate antiviral immune responses, particularly in TNF-α and IFN signaling pathways, while hsa-mir-98-5p[69] is frequently associated with inflammatory responses and cytokine release syndrome, potentially contributing to the pathophysiology of COVID-19. Similarly, the 54 transcription factors identified in our study play essential roles in regulating the transcription of target genes involved in cell growth, differentiation, apoptosis, and immune response. Many of these transcription factors, such as NF-κB, STAT3, and HNF4A, are known to be activated in response to viral infections and inflammation. In the context of COVID-19, for instance, NF-κB is a major regulator of the inflammatory response and has been shown to be activated during SARS-CoV-2 infection, contributing to the cytokine storm that characterizes severe disease[70]. NF-κB plays a critical role in mediating inflammatory responses and regulating pro-inflammatory cytokines, which may influence the immune response to HBV infection and hepatitis B vaccination[71]. In the context of COVID-19, STAT3 plays a critical role in regulating the cytokine storm and immune response, contributing to inflammation. It is activated during infection and drives the recruitment and activation of immune cells, exacerbating the inflammatory response[72]. In HBV, STAT3 modulates immune responses and viral replication, potentially facilitating the progression to chronic infection by influencing immune evasion and viral persistence[73]. HNF4A influences immune responses and cytokine expression in COVID-19, contributing to liver damage and dysfunction[74]. In HBV, HNF4A regulates liver metabolism and viral replication, and its dysfunction may promote chronic infection by altering liver cell homeostasis and facilitating viral persistence[75]. By exploring these miRNAs and transcription factors, we aim to uncover the regulatory networks that drive the gene expression changes observed in both COVID-19 and chronic hepatitis B. Understanding the roles of these upstream regulators will provide valuable insights into the shared and distinct pathological mechanisms of these diseases, offering potential targets for therapeutic intervention and improving our understanding of disease progression.

As modulators of the shared pathogenic process of HBV and COVID-19 disease, the hub genes can be used against both SARS-CoV-2 and hepatitis B virus, potentially providing significant clinical benefits to this patient population. Based on the hub genes, we predicted some drug candidates for the treatment of HBV and COVID-19. Several substances have been evaluated as HBV and COVID-19 therapies. Estradiol reduces susceptibility to COVID-19 and its severity[76]. Estradiol can also trigger a local immune response by activating a large number of cells, such as phagocytes, dendritic cells, natural killer cells, and CD8 T cells. Once these cells are activated, they can fight infection by destroying the SARS-CoV-2, thereby stopping it from spreading to the lower respiratory tract or reducing viral load[77]. Dasatinib can significantly reduce SARS-CoV-2-related mortality, delay its onset, and reduce the number of other clinical symptoms[78]. In addition, dasatinib is a potential therapeutic agent for HBV-associated HCC[49]. Aspirin significantly reduces the risk of HBV-associated HCC[79]. Aspirin use in patients with COVID-19 significantly reduces the risk of fatal course of COVID-19 compared with no aspirin use[80]. Calcitriol is the biologically active form of vitamin D that plays a role in regulating acute and chronic inflammatory responses[81]. Calcitriol inhibits HBV activity by directly targeting the HBV core promoter[82]. Besides, oxygenation was significantly improved in hospitalized COVID-19 patients treated with calcitriol[83].

Despite the valuable insights gained from our bioinformatics and systems biology approach, several limitations must be acknowledged. First, the analysis is based on data retrieved from publicly available datasets, which, while comprehensive, may introduce potential biases related to sample selection, data collection methods, and population differences. These datasets may not fully represent the diversity of patient populations or clinical settings, which could affect the generalizability of our findings. Second, while our analysis was extensive, it remains reliant on computational predictions and pathway enrichment analysis. Third, the heterogeneity across different datasets, such as differences in experimental conditions and batch effects, could also influence the robustness of our results. Finally, the translation of these findings into clinical practice remains a significant challenge, requiring further research to establish the clinical relevance and therapeutic potential of the identified targets, including their effectiveness across diverse populations and clinical contexts.

## Conclusions

This study provides valuable insights into the shared molecular mechanisms underlying COVID-19 and HBV. By identifying common DEGs and hub genes, we have highlighted critical pathways, including immune regulation and cell cycle processes, that may be central to the pathogenesis of both diseases. Additionally, further investigations into the roles of miRNAs and transcription factors could deepen our understanding of the regulatory networks driving disease progression. Furthermore, the study underscores the potential of these shared targets for therapeutic intervention. The identification of drug candidates such as estradiol, dasatinib, and calcitriol opens avenues for potential repurposing in treating both COVID-19 and HBV-related complications. Future research should focus on validating these findings through clinical trials to explore their translational potential. Ultimately, the integration of bioinformatics with experimental approaches will pave the way for more personalized treatment strategies for patients affected by these viral infections.

## Supporting information

S1 Table The differentially expressed genes of HBV. (XLSX)

S2 Table The differentially expressed genes of COVID-19. (XLSX)

S3 Table The common DEGs between HBV and COVID-19. (XLSX)
